# Better than Eating Worms?: Children’s Dietary Exposure to OP Pesticides

**DOI:** 10.1289/ehp.116-a172a

**Published:** 2008-04

**Authors:** Julia R. Barrett

Widespread agricultural use of organophosphate (OP) pesticides frequently leads to low-level exposures in adults and children who eat conventionally grown foods. The frequently used one-time measurement of OP metabolites reveals only short-term exposures, thereby providing little evidence on long-term low-level exposures. A new article presents longitudinal evidence that foods grown in conventional fashion—that is, with the use of pesticides—may be a predominant source of exposure in children **[*EHP* 116:537–542; Lu et al.]**.

The article sprang from the Children’s Pesticide Exposure Study, which focused on two groups of children (3–11 years of age) in the Seattle and Atlanta areas from July 2003 to May 2004. For the present study, the researchers examined 23 children in Seattle (19 of whom completed the study) who normally consumed conventional diets. Parents tracked food consumption during the study and collected urine samples twice daily.

During interventions over the course of the study, the children’s conventional diets were replaced with organic diets and the differences in urinary metabolites of OP pesticides were measured. The organic diets substituted conventionally produced grain, fruit, juice, and vegetables with those produced without pesticides; meat and dairy products rarely contain OP pesticides and were not substituted. During the summer and fall 2003 intervention periods (15 and 12 days, respectively), the children consumed their regular diets from days 1 to 3 and organic diets from days 4 to 8. After day 8, the children resumed their regular diets. There was no organic diet intervention during the winter and spring 2004 sampling periods.

Urine samples were analyzed for metabolites of malathion, chlorpyrifos, diazinon, coumaphos, and methyl pirimiphos; only chlorpyrifos and malathion metabolites were detected frequently enough for statistical analysis. These metabolite levels fell to nearly or fully nondetectable levels within days of the children beginning an organic diet intervention and rose when the children returned to conventional diets.

Given that OP pesticides were reported by parents not to have been used in the home and that urinary metabolites were clearly affected by diet, the researchers conclude that conventionally produced foods were the primary source of OP pesticide exposure for the children in this study. They also attribute higher dietary exposures to imported produce eaten in the winter and spring when domestic produce is not available. This finding is supported by a 2006 Environmental Protection Agency report showing that OP residues on imported produce have risen since 1996 even as residues on domestically grown produce have fallen.

The authors caution, however, that their findings do not promote limiting fresh produce or eating only organic items, as it is unknown whether the observed exposures are harmful. Additionally, the study group did not represent the general population. However, the findings do provide a basis for more accurate assessment of exposure and associated efforts to determine the effects of OP pesticides on children’s health.

## Figures and Tables

**Figure:**
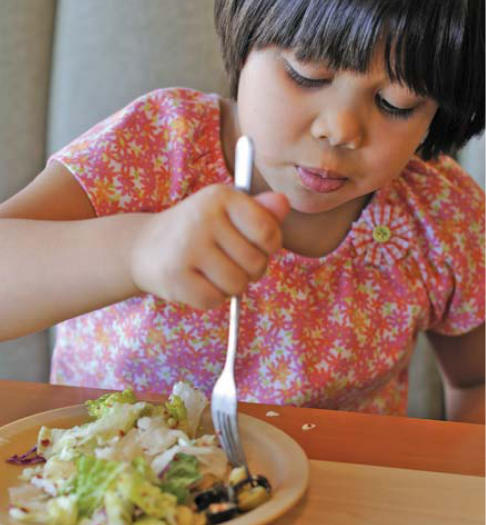
For some families, food is likely the primary source of pesticide exposure

